# DIGITAL LITERACY AND ASSOCIATED FACTORS IN COMMUNITY-DWELLING OLDER ADULTS IN SOUTH KOREA: A QUALITATIVE STUDY

**DOI:** 10.1093/geroni/igac059.2201

**Published:** 2022-12-20

**Authors:** Jiwon Baek, JiYeon Choi, Heejung Kim, Soyun Hong, Yesol Kim, Seongmi Choi, Eunkyung Kim

**Affiliations:** Yonsei University, Seoul, Seoul-t'ukpyolsi, Republic of Korea; Yonsei University College of Nursing, Mo-Im Kim Nursing Research Institute, Seoul, Seoul-t'ukpyolsi, Republic of Korea; Yonsei University, Seoul, Seoul-t'ukpyolsi, Republic of Korea; Yonsei University, Seoul, Seoul-t'ukpyolsi, Republic of Korea; Yonsei University, Seoul, Seoul-t'ukpyolsi, Republic of Korea; Yonsei University, Seoul, Seoul-t'ukpyolsi, Republic of Korea; Yonsei University, Seoul, Seoul-t'ukpyolsi, Republic of Korea

## Abstract

**Background:**

Digital literacy has gained growing importance in the health agenda. However, the level of digital information use and access among Korean older adults is still low at 54%. It is important to understand digital literacy among older adults to provide healthcare and social connectivity, especially during the COVID-19 pandemic. Objective: This study aims to explore the digital literacy and identify barriers to learning and using digital devices among community-dwelling older adults in urban South Korea.

**Methods:**

A qualitative study was conducted using a semi-structured interview guide according to the DigComp 2.0 framework which emphasizes the competencies for full digital participation in five categories: information, communication, content creation, safety, and problem solving. Our sample consisted of 14 older adults (age 68 – 79, 12 women).

**Results:**

Participants reported varying competency of using digital devices for search, communication, and self-management of lifestyle and health. They actively sought help from family and community members to troubleshoot issues related to installation, maintenance, setup software or applications. However, they were passive or evasive in the use of digital devices because of concerns about invasion of privacy or personal information. They struggled to use digital devices owing to physical and cognitive changes associated with aging.

**Conclusion:**

Our findings depict current state and barriers of digital literacy in urban older adults in Korea.

